# Novel Brain-Stiffness-Mimicking Matrix Gel Enables Comprehensive Invasion Analysis of 3D Cultured GBM Cells

**DOI:** 10.3389/fmolb.2022.885806

**Published:** 2022-06-09

**Authors:** Shuowen Wang, Yiqi Wang, Jin Xiong, Wendai Bao, Yaqi Li, Jun Qin, Guang Han, Sheng Hu, Junrong Lei, Zehao Yang, Yu Qian, Shuang Dong, Zhiqiang Dong

**Affiliations:** ^1^ Brain Research Institute, Taihe Hospital, Hubei University of Medicine, Shiyan, China; ^2^ College of Biomedicine and Health, Huazhong Agricultural University, Wuhan, China; ^3^ College of Life Science and Technology, Huazhong Agricultural University, Wuhan, China; ^4^ Department of Neurosurgery, Taihe Hospital, Hubei University of Medicine, Shiyan, China; ^5^ Department of Radiation Oncology, Tongji Medical College, Hubei Cancer Hospital, Huazhong University of Science and Technology, Wuhan, China; ^6^ Department of Thoracic Oncology, Tongji Medical College, Hubei Cancer Hospital, Huazhong University of Science and Technology, Wuhan, China; ^7^ Central Laboratory, Hubei Cancer Hospital, Wuhan, China

**Keywords:** glioblastoma, invasion, tumor multicellular spheroid, high-content imaging, transcriptome analysis

## Abstract

Glioblastoma (GBM) is the most common malignant primary brain tumor in adults, which is fast growing and tends to invade surrounding normal brain tissues. Uncovering the molecular and cellular mechanisms of GBM high invasion potential is of great importance for the treatment and prognostic prediction. However, the commonly used two-dimensional (2D) cell culture and analysis system suffers from lack of the heterogeneity and *in vivo* property of brain tissues. Here, we established a three-dimensional (3D) cell culture-based analysis system that could better recapitulate the heterogeneity of GBM and mimic the *in vivo* conditions in the brain. The GBM cell lines, DBTRG and U251, were cultured by hanging drop culture into the GBM multicellular spheroids, which were embedded in the optimized 3D brain-stiffness-mimicking matrix gel (0.5 mg/ml Collagen Ⅰ + 3 mg/ml Matrigel+ 3.3 mg/ml Hyaluronic Acid (HA)). The biochemical composition of the optimized matrix gel is similar to that of the brain microenvironment, and the elastic modulus is close to that of the brain tissue. The dynamics of the GBM spheroids was examined using high-content imaging for 60 h, and four metrics including invasion distance, invasion area, single-cell invasion velocity, and directionality were employed to quantify the invasion capacity. The result showed that DBTRG cells possess higher invasion capacity than U251 cells, which was consistent with the results of the classic transwell test. Transcriptome analysis of both cell lines was performed to explore the underlying molecular mechanisms. Our novel brain-stiffness-mimicking matrix gel enables comprehensive invasion analysis of the 3D cultured GBM cells and provides a model basis for in-depth exploration of the mechanisms regulating GBM invasion including the interaction between GBM cells and brain stroma.

## Introduction

Glioblastoma (GBM), a WHO grade IV glioma, is one of the most malignant human brain tumors and has a high rate of mortality (Louis et al., 2016; Gramatzki et al., 2017). The current standard treatment for GBM is the maximal lesion excision supplemented by concurrent chemoradiotherapy with temozolomide (TMZ) (Alexander and Cloughesy, 2017). However, GBM tumor tissues are difficult to be completely resected because of their aggressive invasion and infiltrating growth, which leads to recurrence at a later stage. Despite the improvements in therapeutic strategies, the clinical outcomes of GBM patients remain poor (Janjua et al., 2021). Therefore, the dissection of molecular and cellular mechanisms underlying GBM high invasion ability is of great importance for the treatment and prevention of recurrence.

The transwell test with Matrigel coating is commonly used to examine invasion capacity of tumor cells, which uses two-dimensional (2D) monolayer cells cultured on flat and rigid substrates. However, 2D cell culture and analysis cannot restore the tumor heterogeneity, the interaction among tumor cells, and the interaction between tumor cells and the microenvironment. More importantly, in the transwell test of GBM cells, the physical property of the medium such as elastic shear stiffness is much different from that in the brain tissue, so the invasion behavior of GBM cells cannot well recapitulate in *in vivo* conditions. Furthermore, only limited parameters can be obtained in the transwell test, while other aspects of invasion such as the invasion velocity and directionality, which are also important data, but missing. Therefore, it is critical to establish a three-dimensional (3D) culture-based analysis system that can restore the heterogeneity of GBM and simulate the property of the brain tissue to genuinely examine the invasion characteristics of GBM. As a promising method to bridge the gap between cell culture and animal models, various 3D spheroid models have been developed, including hanging drop culture (Foty, 2011), liquid overlay (Costa et al., 2018), spinner cultures (Nath and Devi, 2016), and rotating wall vessel cultures (Hammond and Hammond, 2001). The hanging drop culture is one of the traditional methods and involves the pipetting of cell suspension on a lid, which is then inverted, leading to cell aggregation by surface tension and gravitational force. This method is popularly used due to its simplicity and amenability to high-throughput screening and the size of the spheroid can be adjusted by the initial cell number.

The invasion potential of GBM is regulated not only by its own malignancy but also by the microenvironmental factors such as the stiffness of the surrounding brain tissue. Unlike other solid tumors, GBM infiltratively metastases along the vascular wall and brain parenchyma in most cases (Cuddapah et al., 2014). Anatomical studies have shown that all the vessels in the brain are covered by the basement membrane (Reese and Karnovsky, 1967; Sixt et al., 2001), which is a specialized extracellular matrix with thickness of 50–100 nm and is mainly composed of laminin, fibronectin, and vitronectin (Benton et al., 2014; Cuddapah et al., 2014). On the other hand, the main component of the matrix in the brain parenchyma is hyaluronic acid (HA) (Zimmermann and Dours-Zimmermann, 2008), which is one of the major glucosaminoglycan (GAG) components of the extracellular matrix in the brain and occupies the vast majority of the brain’s extracellular volume (Toole, 2004). The hyaluronic acid-rich microenvironment of the brain promotes cell motility and proliferation of GBM through the receptors for HA, such as CD44 and RHAMM (Toole, 2004, 2009; Sironen et al., 2011). HA is also widely used in biomedical fields due to its favorable biochemical and physical characteristics (Rinaudo, 2008; Muzzarelli, 2011).

To better analyze the invasion characteristics of GBM cells, a brain microenvironment-mimic medium will be needed to embed the cultured tumor multicellular spheroids. Matrigel and Collagen I are currently the most widely used matrix gels (Weiswald et al., 2009; Fayzullin et al., 2019). Matrigel is extracted from the Engelbreth-Holm-Swarm (EHS) mouse tumors, and is composed of four major ECM proteins: laminin, Collagen IV, entactin, and heparan sulfate proteoglycans (Aisenbrey and Murphy, 2020). A series of studies have shown that its composition and structure are similar to that of basement membranes (LeBleu et al., 2007; Benton et al., 2014; Carey et al., 2017). Collagen I is also a major component of the extracellular matrix and is also present in the basement membrane of peritumoral vessels (Giese and Westphal, 1996). Because it contains cell adhesion RGD (Arg-Gly-Asp), which binds to cell surface receptors, Collagen I, derived from animal tissues, is also a commonly used scaffold in cell culture and tissue engineering. Tumor cells proliferate, migrate, and infiltrate in Collagen I by the secreting collagen cleavage enzymes to remodel them (Ala-aho and Kähäri, 2005). Previous studies have used Collagen I + Matrigel hybrid gel to simulate the brain tissue property (Berens et al., 2015; Fayzullin et al., 2019; Dundar et al., 2020), but some parameters such as the stiffness are very different from that of the brain tissue (Levental et al., 2007; Anguiano et al., 2017; Chaudhuri et al., 2020).

This study aims to establish an optimized *in vitro* 3D culture and analysis system to test GBM cell invasion. Using GBM cell lines, DBTRG and U251, we first explored the conditions for culturing GBM multicellular spheroids by the hanging drop methods. Next, the brain invasion analysis medium was optimized by adding HA to the Collagen I + Matrigel hybrid gel, which we termed as brain-stiffness-mimicking (BSM) matrix gel. The biochemical composition and elastic modulus of the optimized BSM matrix gel were proved to be similar to that of the brain extracellular matrix. We also proposed a quantification method to examine multiple parameters of the invasion behavior of GBM cell spheroids. Finally, the invasion capacity of DBTRG and U251 cell lines was verified by the classical transwell test and the underlying mechanisms were investigated by transcriptomic analysis. Our system provides a useful platform to test the comprehensive invasion behavior of the glioma cells in a brain extracellular matrix-mimicking medium, which will facilitate the mechanistic study and the development of novel therapy of GBM.

## Materials and Methods

### Reagents

DMEM (SH30243.01) and RPMI 1640 (SH30809.01) medium were purchased from GE HyClone (Utah, United States); Matrigel (354234) from Corning (New York, United States); Collagen I, rat tail (A10483-01) from Gibco (CA, United States); HA (20153641157) from Changzhou Institute of Materia Medica Co., Ltd (Changzhou, China); Trypsin-EDTA (BL512A) from Biosharp (Hefei, China); Fetal Bovine Serum (C04001-500) from VivaCell (Shanghai, China); Rabbit anti-HIF-1α (20960-1-AP) from San Ying Biotechnology (Wuhan, China); Mouse anti-Ki-67 (9449) from the Cell Signaling Technology (Shanghai, China); Penicillin–Streptomycin Solution (BL505A) from Shanghai Qiaoxing Trading Co., Ltd. (Shanghai, China); and Methyl cellulose (M8070), Calcein-AM (C7600), and Propidium Iodide (C0080) from Solarbio (Beijing, China).

### Hanging Drop Culture of Glioblastoma Multicellular Spheroids

The GBM multicellular spheroids were established by the hanging drop culture method. The cell suspensions were diluted to 2,500 cell/20 μl, 5,000 cell/20 μl, 10,000 cell/20 μl, and 20,000 cell/20 μl in RPMI (DBTRG) or DMEM (U251) medium containing 0.24% methyl cellulose. 20 μl of cell suspension was slowly transferred to the center of the each well of the 24-well plate, followed by inverting the 24-well plate cover and adding 5 ml of PBS buffer to the cover to prevent the evaporation of the cell culture medium. Then, slowly turning the 24-well plate upside down. The cell suspensions were cultured in the incubator at 37°C and 5% CO_2_. The tumor multicellular spheroids were imaged at 12 h intervals over a 72-h period.

### Calcein-AM/Propidium Iodide Staining Assay of Glioblastoma Multicellular Spheroids

The GBM multicellular spheroids were collected at day 1, 4, and 7 after spheroid formation, and stained with Calcein-AM/PI for the live and dead cells, respectively. The GBM multicellular spheroids were first placed in the center of a confocal dish, followed by the addition of 100 μl of PBS buffer and 10 μl of 2 µM of Calcein-AM and PI and blown and mixed. They were then incubated for 30 min at room temperature and protected from light. The staining of the GBM multicellular spheroids was imaged under a Revolution WD spinning confocal disk (Andor Technology, United Kingdom).

### Cryosectioning of Glioblastoma Multicellular Spheroids

The GBM multicellular spheroids were first rinsed in PBS wash buffer on day 4 after spheroid formation and then transferred to 4% formaldehyde for 6 h. After fixation, the spheroids were sequentially dehydrated with freshly prepared 15% and 30% sucrose solutions until the spheroids sink to the bottom. After dehydration, the GBM multicellular spheroids were embedded in the OCT blocks and then frozen in a refrigerator at -80 °C for 4 h. The GBM spheroid blocks were sectioned at a thickness of 10 µm. The sections were collected on a glass slide and air-dried in a biosafety hood for 6 h followed by immunostaining immediately afterward or were stored at –20°C.

### Immunostaining of Glioblastoma Spheroid Cryosections

The sections were washed three times with PBS for 10 min each, and then permeabilized in PBS+0.5% Triton X-100 for 10 min, followed by triple-wash in PBS for 10 min each. The sections were then incubated in blocking solution at room temperature for 1 h. After blocking, the sections were incubated with HIF-1α and KI-67 primary antibodies (1:500 in PBS) overnight at 4°C in a dark box. The sections were washed four times with PBS for 10 min each. Then, 1:500 diluted secondary antibodies against rabbit or mouse were added to the sections and incubated for 2 h at room temperature. The sections were washed four times with PBS for 10 min each and then incubated with Hoechst solution for 10 min at room temperature. Finally, after washing with PBS three times, the sections were sealed and imaged.

### Preparation of 3D Matrix Gel for Invasion Analysis of Glioblastoma Multicellular Spheroids

(1) Matrix gel without HA

Collagen I and Matrigel were thawed and stored on ice in advance, and the subsequent steps were performed on ice. 3 mg/ml of Collagen I was diluted to 1 mg/ml using 10×PBS, 1 mol/L NaOH, and dH2O. 10 mg/ml of Matrigel substrate gel was diluted to 6 mg/ml with RPMI or DMEM serum-free medium, respectively. 1 mg/ml Collagen I and 6 mg/ml Matrigel were mixed in equal volumes to obtain the matrix gel with 0.5 mg/ml Collagen I + 3 mg/ml Matrigel.

2) Matrix gel with HA

1.5 mg/ml Collagen I and 9 mg/ml Matrigel were prepared according to the above protocol. 1.5 mg/ml Collagen I, 9 mg/ml Matrigel, and 10 mg/ml HA were mixed in equal volumes to obtain the matrix gel with 0.5 mg/ml Collagen I + 3 mg/ml Matrigel +3.3 mg/ml HA.

### Measurement of the Elastic Modulus of 3D Matrix Gels

The matrix gels of 0.5 mg/ml Collagen I + 3 mg/ml Matrigel and 0.5 mg/ml Collagen I + 3 mg/ml Matrigel +3.3 mg/ml HA were prepared according to the abovementioned protocols. Then, the oscillatory shear rheological measurement was performed in the parallel-plate configuration using a 20 mm diameter top plate. 50 μl gel solution was placed between the parallel-plates of the rheometer, and the distance between the plates was set to 0.1 mm. The *in-situ* crosslinking was performed at 37°C for 30 min. After crosslinking, the amplitude sweeps were performed at a constant frequency to determine the linear viscoelastic range of each sample. Then, the elastic modulus was determined by the frequency sweeps in the linear range with the frequencies from 0.05 to 100 Hz at a strain amplitude of 1.5%.

### Quantification of the Invasion Capacity of Glioblastoma Multicellular Spheroids in 3D Matrix Gel

To evaluate the invasion capacity, the GBM multicellular spheroids were embedded in the matrix gel (with HA or without HA) in a 96-well plate and imaged under a Cytation five high-content cell imaging system with built-in cell culture module from BioTeck, with 30-min intervals for 60 h. Subsequently, analysis of invasion distance, increased invasion area, single cell invasion velocity, and directionality in the matrix gel were analyzed using ImageJ imaging software (NIH ImageJ) and Chemotaxis and Migration Tool (ibidi).

### Transwell Cell Invasion Assay

Matrigel was diluted 10-fold using serum-free RPMI or DMEM medium, and 50 μl of Matrigel was transferred to the top of the 8 μm filter membrane in a transwell insert and left overnight in the cell incubator until the Matrigel solidified. On the next day, 50 μl of the serum-free RPMI or DMEM medium were added to the filter the membrane and maintained for 30 min. Cell suspension was prepared with cell density adjusted to 2.0×10^5^/ml in serum-free cell culture medium containing 0.1% BSA (bovine serum albumin) and 200 μl cell suspension was added to the top of the filter membrane, while 500 μl RPMI or DMEM medium to the lower chamber, which was followed by 48 h incubation. Finally, the cells in the filter membrane were fixed with 4% PFA, stained with 0.2% crystal violet, and photographed under a microscope, and the number of traversing cells in the lower chamber was counted using ImageJ software.

### Transcriptome Analysis

Quality control of the raw RNA-seq sequences was initially performed according to Fastp application. Fastp can deal with both trimming and quality controlling to ensure high quality reads exercise in the following formal analysis. The application in reads alignment procedure was Hisat2, whose reference genome was hg38 with the default parameters in our paired reads. HTSeq package was used to construct count matrix with mapping results. Then, the DESeq2 built a model with the observed counts for differential expression analysis with a threshold log2FC>±2 and *p*-value<0.05. These differentially expressed genes (DEG) were enrolled in the following enrichment and GSEA analysis through the ClusterProfiler package.

Enrichment functions of the corresponding GO process, KEGG, and REACTOME pathway were called with the filtration cutoff values as both *p*-value and Q-value (P-adjust) less than 0.05. Then, the plotting built-in function had been invoked for dotplots and heatmaps presentation.

### Statistical Analysis

GraphPad Prism 9 software was used for statistical analysis, and the results were presented as the mean with Standard Error of Mean (SEM). The significance of differences between the different groups was analyzed by Student’s t-test, *p* < 0.05 was considered statistically significant.

## Results

### Culture Condition Optimization and Growth Kinetics Analysis of Multicellular Glioblastoma Spheroids

**FIGURE 1:**
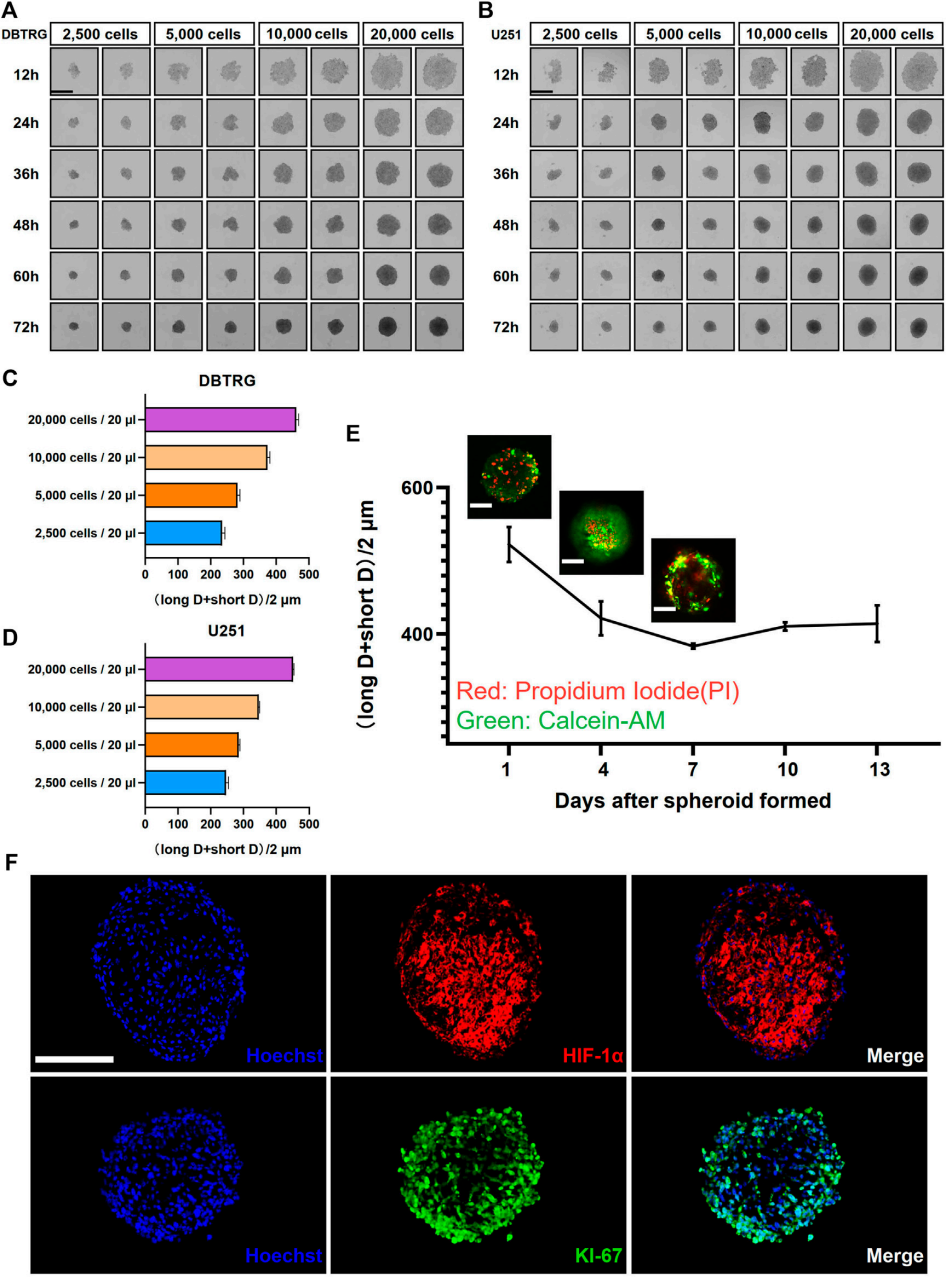
Glioblastoma multicellular spheroid-forming parameters and their internal heterogeneity. Representative images show the formation of DBTRG **(A)** and U251 **(B)** tumor multicellular spheroids at various spheroidization concentrations. Scale bar = 500 µm. Bar graphs show the diameters of DBTRG **(C)** and U251 **(D)** Tumor multicellular spheroids generated at 72 h under different spheroid formation concentrations. **(E)** Growth kinetics of U251 tumor multicellular spheroids and the distribution of live and dead cells. Scale bar = 200 µm. **(F)** Immunofluorescence staining against HIF-1α and KI-67 on cryosections of tumor multicellular spheroids at day 4 after spheroid formation. Scale bar = 200 µm.

We employed the GBM cell lines, DBTRG and U251, to investigate the optimal culture conditions for generating tumor multicellular spheroids. The GBM multicellular spheroids were cultured by the hanging drop culture method for 72 h at four starting concentration gradients of 2,500 cells/20 μl, 5,000 cells/20 μl, 10,000 cells/20 μl, and 20,000 cells/20 μl. We determined that the tumor multicellular spheroids were best generated at the initial concentration of 20,000 cells/20 μl, which resulted in reasonable sized tumor spheroids (461.41 ± 7.06 µm in diameter for DBTRG; 451.45 ± 2.45 µm in diameter forU251) and recapitulated the tumor heterogeneity with a necrotic core (Figures 1A–D). The multicellular GBM spheroids showed dynamic growth as the size changed over time during the long-term culture (Supplementary Figures S1A,B). We next examined the growth kinetics of U251 multicellular spheroids in the hanging drop culture method for 13 days. We measured the orthogonal diameter of tumor spheroids at day 1, 4, 7, 10, and 13 after spheroid formation (Figure 1E). Staining with Calcein-AM and PI was performed to show the live cells (green) and dead cells (red) at day 1, 4, and 7 (Figure 1E). The results showed that the size of the tumor multicellular spheroids decreased first, then increased, and tended to stabilize within 13 days. The shortest diameter was observed on day 7, but the diameter of the spheroids was still longer than 400 μm at that time. On day 4, dead cells started to appear inside the spheroids. The number of dead cells gradually increased at the core of the spheroids from day 1 to day 7. On day 7, live cells were mainly concentrated in the outer area of the spheroids (Figure 1E). To examine the cellular heterogeneity, we performed the immunofluorescence staining of the hypoxic marker HIF-1α and proliferation maker KI-67 on frozen sections of the GBM multicellular spheroids at day 4. The results showed that hypoxic cells were concentrated in the center, while proliferating cells were enriched in the outer layer (Figure 1F).

### Cell invasion Analysis of Glioblastoma Multicellular Spheroids in 0.5 mg/ml Collagen I + 3 mg/ml Matrigel

**FIGURE 2:**
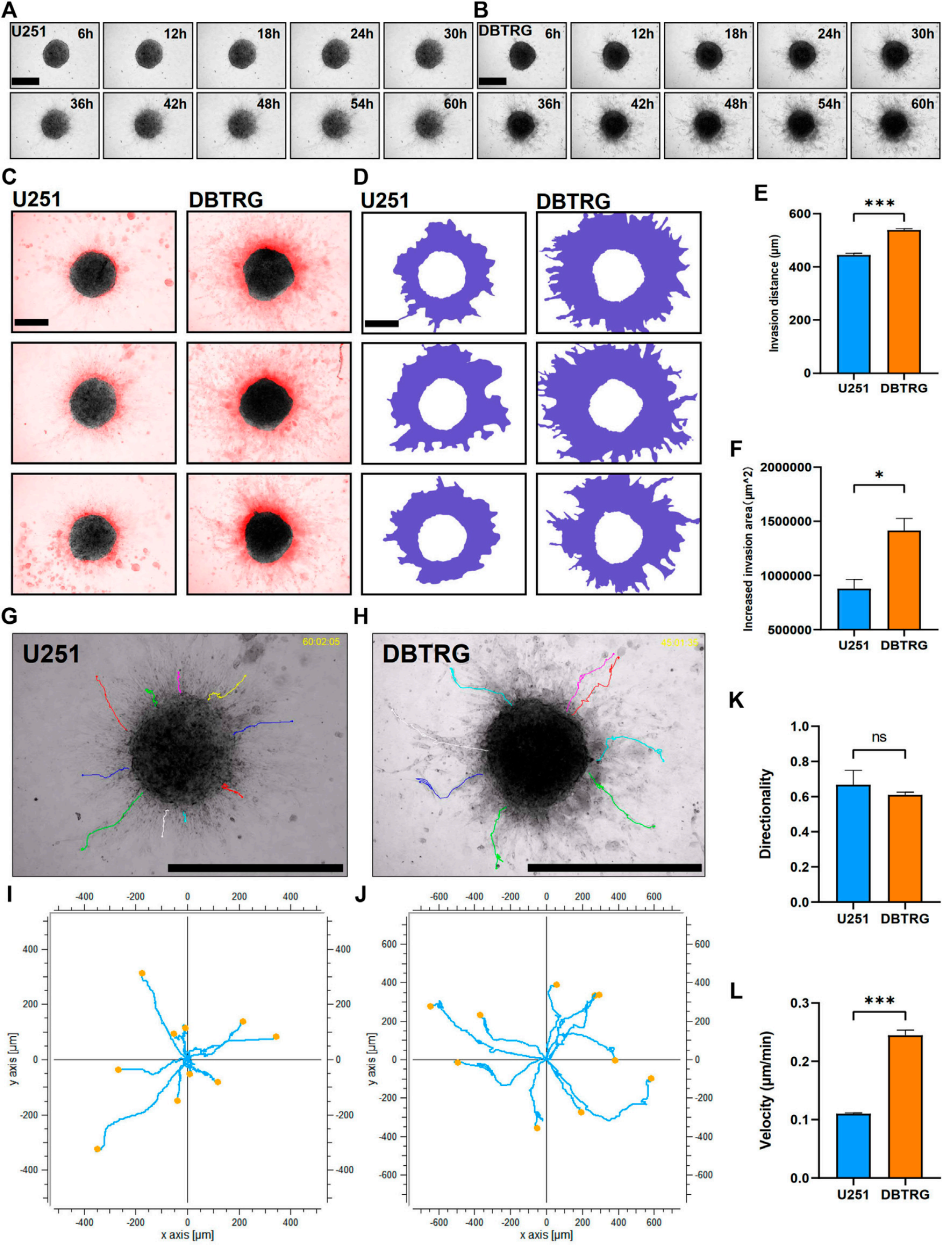
Quantitative invasion analysis of DBTRG and U251 spheroid in 0.5 mg/ml Collagen Ⅰ + 3 mg/ml Matrigel. **(A,B)** Representative images of U251 and DBTRG spheroids in 0.5 mg/ml Collagen Ⅰ + 3 mg/ml Matrigel over a 60-h period. Scale bar = 600 µm. **(C)** Invasion of U251 and DBTRG tumor multicellular spheroids in 0.5 mg/ml Collagen Ⅰ + 3 mg/ml Matrigel for 60 h. The black color is the distribution of the tumor multicellular spheroid at 0 h, and the red color indicates the distribution of the tumor multicellular spheroid at 60 h minus that of 0 h. Scale bar = 400 µm. **(D)** Invasion area (purple area) of U251 and DBTRG spheroids within 60 h in 0.5 mg/ml Collagen Ⅰ + 3 mg/ml Matrigel. Scale bar = 400 µm. Statistical data show the difference of invasion distance **(E)** and invasion area **(F)** between the U251 and DBTRG spheroids. **(G,H)** The merged image of single cell trajectory and spheroid. Scale bar = 1000 µm. **(I,J)** Trajectory of representative single cells in the U251 and DBTRG spheroids. Statistical data show the difference of invasion directionality **(K)** and invasion velocity **(L)** between the cells of U251 and DBTRG spheroids. *p < 0.05, ***p < 0.001, ns: no significance.

We next examined and analyzed the invasion capacity of GBM cells from the multicellular spheroids using 3D matrix gels with the composition similar to the published ones. Briefly, the GBM multicellular spheroids were removed and embedded in matrix gel with 0.5 mg/ml Collagen I + 3 mg/ml Matrigel. We performed high-content live imaging for 60 h to quantify the invasion capacity of tumor cells (Figures 2A,B). We quantified multiple parameters of the invasion behavior and the results showed that the invasion distance and invasion area of the DBTRG tumor multicellular spheroids were significantly higher than those of U251 (Migration distance: 539.02 ± 5.09 µm vs. 444.88 ± 6.63 µm; Increased migration area: 1,415,171.33 ± 111,834.89 µm^2^ vs. 878,177.66 ± 84,423.97 µm^2^) (Figures 2C–F). We further analyzed the invasion capacity of single cells. The average directionality of the invading single cells from the DBTRG spheroids was not significantly different from that of U251 (0.61 ± 0.02 vs. 0.69 ± 0.08), while the invasion velocity of DBTRG single cells was significantly higher than that of U251 (0.24 ± 0.01 μm/min vs. 0.11 ± 0.002 μm/min) (Figures 2G–L). The results, obtained from our 3D culture and analysis system using regular matrix gel, indicated that the cells of DBTRG tumor multicellular spheroids gained more robust invasion capacity than those of U251.

### Optimization of 3D Brain-Stiffness-Mimicking Matrix Gel

**FIGURE 3:**
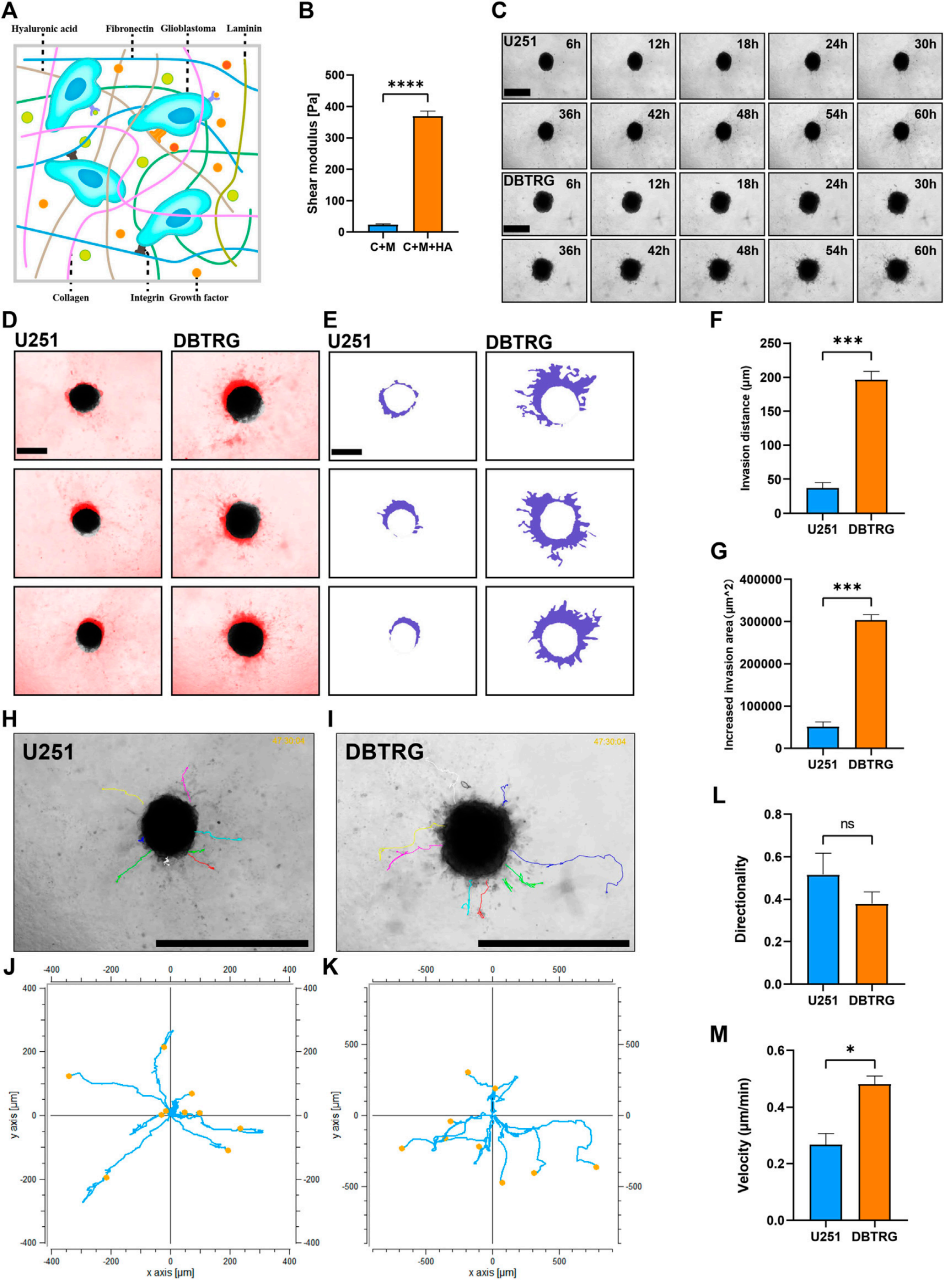
Quantitative invasion analysis of DBTRG and U251 spheroid in 0.5 mg/ml Collagen Ⅰ + 3 mg/ml Matrigel +3.3 mg/ml HA. **(A)** Schematic shows the matrix proteins enriched in the brain microenvironment. **(B)** The shear elastic modulus of the matrix gel increased from 23.04 ± 2.84 Pa to 367.91 ± 17.43 Pa after adding hyaluronic acid to the matrix gel of Collagen Ⅰ + Matrigel. **(C)** Representative images of U251 and DBTRG spheroids in 0.5 mg/ml Collagen Ⅰ + 3 mg/ml Matrigel +3.3 mg/ml HA within 60 h. Scale bar = 600 µm. **(D)** Invasion of U251 and DBTRG tumor multicellular spheroids in 0.5 mg/ml Collagen Ⅰ + 3 mg/ml Matrigel +3.3 mg/ml HA within 60 h. The black color is the distribution of the tumor multicellular spheroid at 0 h, and the red color indicates the distribution of the tumor multicellular spheroid at 60 h minus that of 0 h. Scale bar = 400 µm. **(E)** Invasion area (purple area) of U251 and DBTRG spheroids within 60 h in 0.5 mg/ml Collagen Ⅰ + 3 mg/ml Matrigel +3.3 mg/ml HA. Scale bar = 400 µm. Statistical data show the difference of invasion distance **(F)** and invasion area **(G)** between the U251 and DBTRG spheroids. **(H,I)** The merged image of single cell trajectory and spheroid. Scale bar = 1000 µm. **(J,K)** Trajectory of representative single cells in U251 and DBTRG spheroids. Statistical data show the difference of invasion directionality **(L)** and invasion velocity **(M)** between the cells of U251 and DBTRG spheroids. *p < 0.05, ***p < 0.001, ****p < 0.0001, ns: no significance.

Biophysical factors, in the vicinity of the tumor, can have a strong impact on tumor cell behavior including invasion. HA is the most abundant extracellular matrix component in the brain parenchyma and may influence the behavior of GBM cells by interacting with the receptors on the cell surface (Figure 3A). Therefore, we supplemented HA to 0.5 mg/ml Collagen I + 3 mg/ml Matrigel to mimic the stiffness of the brain tissue. The elastic modulus of the matrix gel, which is the parameter of the elastic shear stiffness, increased from less than 50 Pa (23.04 ± 2.84 Pa) to nearly 370 Pa (367.91 ± 17.43 Pa) (Figure 3B), conforming to the reported range of the elastic modulus of the normal brain (Levental et al., 2007; Budday et al., 2017; Weickenmeier et al., 2018; Axpe et al., 2020; Chaudhuri et al., 2020). On the other hand, the biochemical composition of the brain matrix is better restored as HA is the main component of the brain parenchymal matrix. We, therefore, successfully established the 3D BSM matrix gel for *in vitro* analysis of GBM cell invasion.

### DBTRG Cells Showed Higher Invasion Capacity Than U251 Cells in BSM Matrix Gel

We next examined and quantified the invasion capacity of DBTRG and U251 multicellular spheroids in the BSM matrix gel using high-content imaging for 60 h (Figure 3C). The results showed that the invasion distance and invasion area of DBTRG spheroids were also significantly higher than those of U251 in the BSM matrix gel (migration distance: 196.42 ± 12.52 µm vs. 36.89 ± 8.45 µm; increased migration area: 303,217.67 ± 13,240.97 µm^2^ vs. 51,631.67 ± 11,198.06 µm^2^) (Figures 3D–G). The average invasion directionality of the single cells from DBTRG spheroids was not significantly different from that of U251 (0.38 ± 0.06 vs. 0.52 ± 0.1), while the invasion velocity of the single cells of DBTRG was significantly higher than that of U251 (0.48 ± 0.03 μm/min vs. 0.27 ± 0.04 μm/min) (Figures 3H–M).

Thus, consistent with the results obtained using the regular matrix gel, our quantitative analysis of the invasion capacity of the GBM multicellular spheroids using the optimized BSM matrix gel indicated that the invasion capacity of cells in the DBTRG spheroids was significantly higher than that of U251.

### DBTRG Cells Showed Higher Invasion Capacity Than U251 Cells in 2D Culture

**FIGURE 4:**
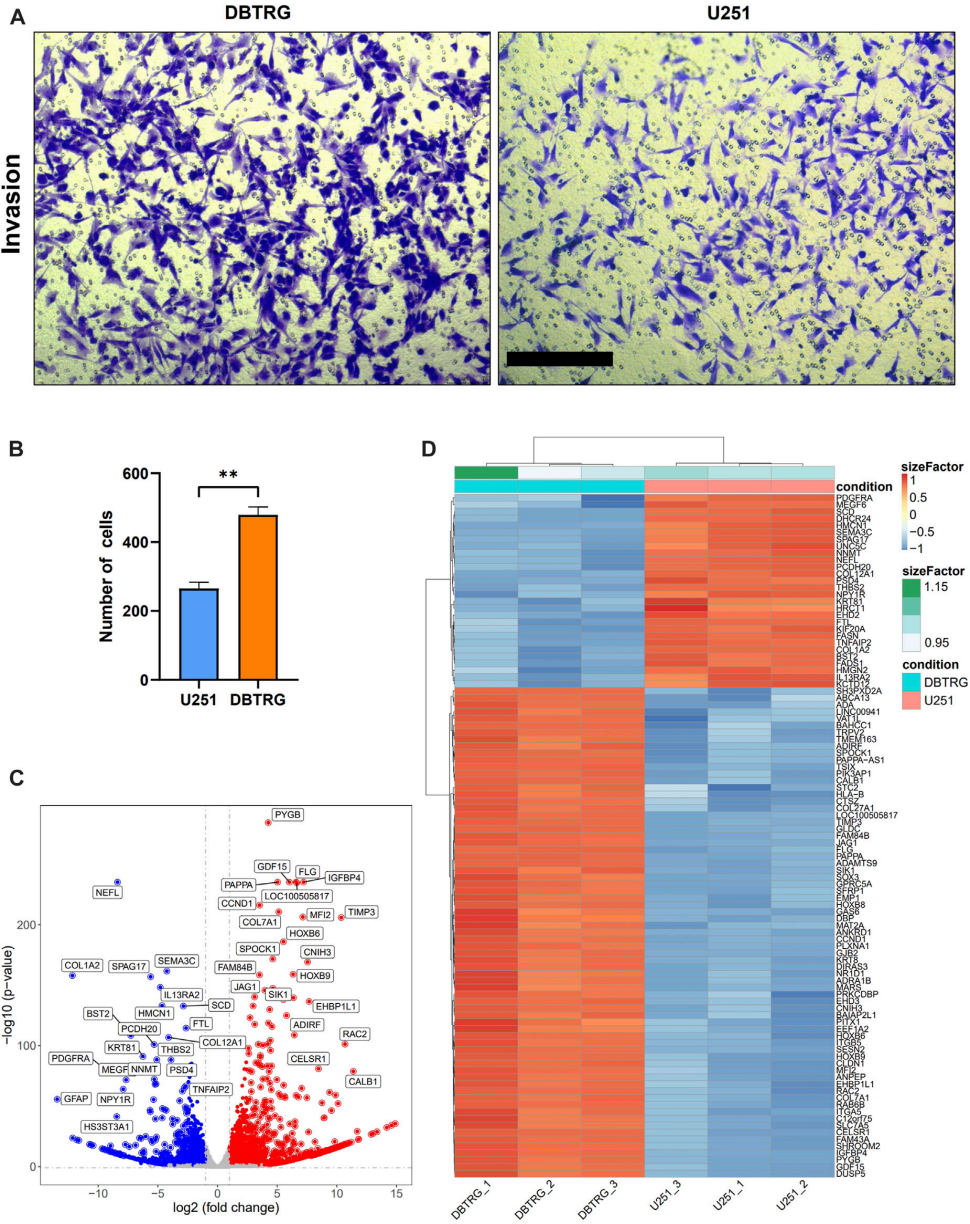
Comparison of the invasion capacity and summary of DEGs between DBTRG and U251. **(A)** Representative images of the transwell invasion assay. Scale bar = 200 µm. **(B)** Statistical results of the transwell invasion assay. **p < 0.01. **(C)** Volcano plot of the DEGs in DBTRG and U251 cells. **(D)** Heat map shows top DEGs in DBTRG and U251 cells.

We examined the invasion capacity of 2D-cultured DBTRG and U251 cells using the classic transwell assay with Matrigel chamber. The results showed that the number of cells in the DBTRG group that invaded through the chamber (479.33 ± 22.67) was significantly higher than the number of cells in the U251 group (265.33 ± 18.44) (Figures 4A,B). Therefore, 2D culture assay further verified the conclusion of the tumor spheroid invasion study using our 3D culture and analysis system with the optimized BSM matrix gel.

### Transcriptomic Analysis Revealed Gene Expression Differences Between DBTRG and U251 Cells

In order to explore the molecular mechanisms underlying the different invasion ability of DBTRG and U251 cells, we performed transcriptomic analysis to examine the gene expression differences between these two GBM cell lines. DBTRG and U251 cells in 2D culture were harvested for RNA-seq. The differentially expressed genes (DEGs) were filtrated under the condition of both *p*-value < 0.05 and [log2FoldChange (log2FC)] > 1 (Figure 4C). A total of 2774 DEGs were identified, among which 1,499 genes were highly expressed and 1,275 genes were lowly expressed in DBTRG compared to U251. The top 100 DEGs were shown in the heatmap (Figure 4D) and the details of all the DEGs could be found in Supplementary Table S1.

**FIGURE 5:**
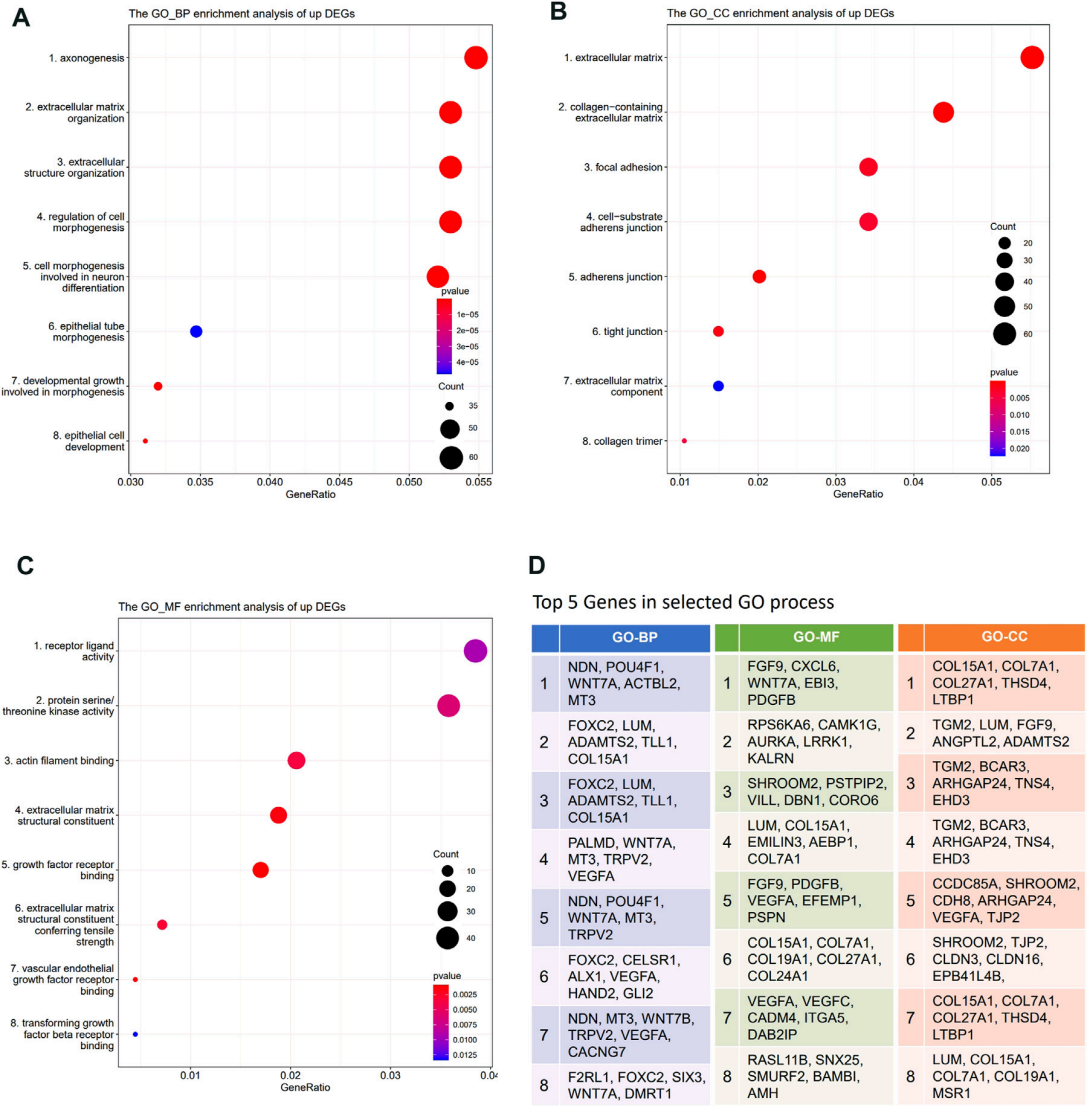
Transcriptome analysis of DEGs between DBTRG and U251. **(A)** GO_BP enrichment analysis of the upregulated genes in DBTRG. **(B)** GO_CC enrichment analysis of the upregulated genes in DBTRG. **(C)** GO_MF enrichment analysis of the upregulated genes in DBTRG. **(D)**Top five genes in the selected GO terms.

Gene Ontology (GO) enrichment analysis, KEGG, and REACTOME pathway enrichment analysis were performed on the DEGs. The results showed that the lowly expressed DEGs in DBTRG were enriched in chromosomes, immune, and disease-related pathways, such as chromosome segregation, IL−17 signaling pathway, and lipid and atherosclerosis pathway (Supplementary Figure S2), which are not directly correlated with tumor invasion. On the other side, GO enrichment analysis showed that the highly expressed DEGs were enriched in GO terms that correlated with the extracellular matrix, cell junction, and cell motility, which are important factors for regulating tumor invasion. Specifically, the highly expressed DEGs were enriched in biological processes (BP) such as axonogenesis, extracellular matrix organization, extracellular structure organization, and regulation of cell morphogenesis (Figure 5A, Supplementary Figure S3A); molecular functions (MF) such as receptor ligand activity, actin filament binding, and extracellular matrix structural constituent (Figure 5B, Supplementary Figure S3B); cellular components (CC) such as extracellular matrix, collagen-containing extracellular matrix, cell-substrate adherents junction, and collagen trimer (Figure 5C, Supplementary Figure S3C). We summarized the top five genes in these GO terms (Figure 5D), of which many have been proved to play an important role in tumor invasion. For example, our data revealed that *WNT7A*, *VEGFA*, *EFEMP1*, *TRPV2,* and *FOXC2* were significantly highly expressed in DBTRG, which was likely one of the molecular bases for the stronger invasion capacity of DBTRG, as previous studies have proved that upregulation of these genes could enhance the invasion ability of tumor cells (Li et al., 2013; Gong et al., 2014; Wang et al., 2015; Liu and Liu, 2018; Kato et al., 2022). Furthermore, KEGG and REACTOME enrichment analysis also demonstrated that the highly expressed DEGs in DBTRG mainly concentrated in the pathways that correlated with tumor invasion, e.g., ECM−receptor interaction, extracellular matrix organization, degradation of the extracellular matrix, collagen formation, collagen biosynthesis and modifying enzymes, crosslinking of collagen fibrils, collagen chain trimerization, and signaling pathways such as MAPK and mTOR pathways (Supplementary Figures S4A,B). The raw sequencing data could be found in Gene Expression Omnibus (GEO) with the accession number GSE200574 (https://www.ncbi.nlm.nih.gov/geo/query/acc.cgi?acc=GSE200574).

## Discussion

GBM is the primary brain tumor with the worst prognosis (Gramatzki et al., 2017). Due to the aggressive invasion of GBM cells, GBM tends to grow infiltratively to invade the surrounding brain tissues including important functional areas, which usually causes severe symptoms such as headache, vomiting, and cerebral edema. Also, because of the diffused invasion property, GBM is difficult to be resected completely by surgery and is prone to relapse. To inhibit the GBM cell invasion is, thus, one of the keys to treat this devastating disease, which relies on further understanding of the cellular and molecular mechanisms that regulate the invasive competency of GBM cells. The commonly used 2D culture invasion assay such as transwell test with Matrigel coating could not recapitulate the *in vivo* property of the brain microenvironment including physical resistance as well as brain-specific extracellular matrix. Therefore, comprehensive 3D models that mimic the brain microenvironment, coupled with comprehensive analyses, are critical for the study of GBM invasion. In this study, we established a novel *in vitro* 3D culture and analysis system, and provided a model basis for the study of GBM invasion. Our model system will facilitate the dissection of molecular basis for GBM progression and recurrence, and lay foundation to discover novel targets that can be used to develop effective therapeutic strategy.

We cultured DBTRG and U251 cells in the hanging drops for 3 days at a concentration of 20,000 cells/20 μl to generate GBM multicellular spheroids with a diameter of 400–500 µm. Calcein-AM/PI staining assay showed that a necrotic core started to appear inside the spheroid on the fourth day after spheroid formation, and gradually increased to occupy larger area inside the spheroid as the culture time increased. Immunostaining against hypoxia marker HIF-1α and proliferation marker KI-67 revealed that the degree of hypoxia was significantly higher in the inner cells than the outer cells at day 4 after spheroid formation, while the proliferation potential of the outer cells was significantly higher than the inner cells, consistent with the gradient of hypoxia and proliferation in the solid tumors. We have successfully generated the GBM multicellular spheroids that recapitulate the key features of glioblastoma: necrotic core, hypoxia, and proliferation gradient. However, it is worth exploring other spheroid culturing methods as the drug perturbations are not directly feasible with the hanging drop culture method.

There is an emerging consensus that the elasticity or stiffness of the ECM plays a critical role in a variety of biological processes and the mechanical features of the ECM can have a potent impact on tumor progression (Butcher et al., 2009; Kumar and Weaver, 2009). For example, the morphology and proliferation of GBM cells cultured on 2D polyacrylamide matrix were regulated by the ECM stiffness (Sen et al., 2009; Ulrich et al., 2009), and the morphology and motility of GBM cells cultured in 3D hydrogels could also be altered with the changes in hydrogel stiffness (Ananthanarayanan et al., 2011). The mammalian brain is a relatively soft tissue that exhibits a complex set of mechanical characteristics. Previous studies have shown that the elastic modulus of the brain is around 100 Pa-2000 Pa (Levental et al., 2007; Budday et al., 2017; Weickenmeier et al., 2018; Axpe et al., 2020; Chaudhuri et al., 2020). The commonly used *in vitro* matrix gels are Matrigel (Aisenbrey and Murphy, 2020), Collagen I (Ala-aho and Kähäri, 2005), and other gel materials that can gelate spontaneously or by adding crosslinking agents. However, the mechanical features of the previously published matrix gels, especially stiffness, is much different from the brain tissue (Anguiano et al., 2017). The combination of Matrigel and Collagen I mimic the major components of the extracellular basement membrane of the brain blood vessels, but lacks HA, a nonsulfate glycosaminoglycan, which is the major component of the brain parenchyma (Bignami et al., 1993; Yasuhara et al., 1994). In order to establish an optimized 3D matrix gel for GBM invasion analysis, we combined HA with the mixed gel of Collagen I and Matrigel. The final concentrations of Collagen I and Matrigel are 0.5 and 3 mg/ml, respectively, which are in accordance with the invasion microenvironment of GBM in the brain. By adjusting the amount of HA, the elastic modulus of the matrix gel was made close to the actual character of the brain tissue.

We performed high-content imaging on the GBM spheroids embedded in both Collagen I/Matrigel mixed gel and our optimized BSM matrix gel with HA, from which we obtained multiple parameters including migration distance, increased migration area, single cell migration velocity, and directionality to analyze the invasion capacity of GBM cells. We found that DBTRG had a stronger invasion capacity than U251 in both the matrix gels, which is further confirmed by the classical transwell test, indicating that our 3D culture and analysis system can reliably assess GBM cell invasion capacity with more quantitative parameters in a 3D microenvironment that more genuinely reflects the *in vivo* property of the brain.

In order to explore the molecular mechanisms of the invasion difference between DBTRG and U251, we profiled the DEGs in these two GBM cell lines using the transcriptomic analysis. Some DEGs have been proved to play an important role in regulating tumor cell invasion ability such as *WNT7A*, *VEGFA*, *EFEMP1*, *TRPV2,* and *FOXC2*, whose expressions are consistent with the phenotype (see Results). We also found some significantly upregulated DEGs in DBTRG that have strong correlation with DBTRG high invasion capacity, while have not been previously reported to be involved in tumor invasion. For example, the metalloproteases gene *ADAMTS2* has been proved to be highly expressed in GBM stem cells (Cheng et al., 2011), while no direct evidence has shown that this gene can increase GBM invasion. ADAMTS2 is a member of the “A Disintegrin And Metalloproteinase with ThromboSpondin type I domain” proteases (ADAMTS) family, which ensures the assembly of precollagen trimers into collagen fibers by cleaving the amino-propeptide (Bekhouche and Colige, 2015). The ADAMTS family proteases play important roles in the proteolytic degradation of the extracellular matrix during cell invasion (Takahashi et al., 2014). A later study in gastric cancer also showed that the upregulated *ADAMTS2* expression was relevant to poor prognosis (Jiang et al., 2019). This gene could be a novel key molecule underlying the higher invasion capacity of DBTRG and could potentially serve as a novel therapeutic target of GBM.

Compared with the other 3D model, our culture-based analysis system has several advantages. The biochemical composition and stiffness of the brain-stiffness-mimicking matrix gel are close to the brain and the hanging drop culture method is simple and inexpensive with good compatibility, which allows coculture of multiple cells and real-time imaging of the cellular invasion process. Furthermore, quantitative analysis of multiple invasion parameters enables the multifaceted characterization of the invasive capacity of glioma cells. In addition to analyzing the invasion capacity, our system can be applied under multiple scenarios. For example, cell spheroids can be kept in the hanging drop culture to quantify the proliferation ability of different glioma cells by comparing the spheroid size. It is also possible to apply radiation or drugs to the tumor spheroids to explore mechanisms underlying resistance of glioma to radiation or chemical therapy. On the other hand, GBM primary cells for spheroid culture and invasion experiments will better restore tumor heterogeneity, interactions between tumor cells, and interactions between tumor cells and the microenvironment. Therefore, the advantage of the optimized 3D model can be better reflected by using GBM primary cells for spheroid culture.

The 3D culture and analysis system can be subsequently optimized from two perspectives. On one hand, besides GBM cells, multiple cell types such as astrocytes, endothelial cells, and microglia, can be mixed for the 3D culture of multicellular spheroid to better restore the GBM microenvironment *in vitro*. On the other hand, animal-derived decellularized brain matrix, new microfluidics, or 3D cell printing techniques can be used for the invasion analysis, which might better recapitulate the mechanical features of the brain tissues in certain aspects.

## Conclusion

In this study, we explored the optimal conditions for culturing the GBM multicellular spheroids using the hanging drop method, and examined the growth kinetics as well as the internal heterogeneity of the GBM spheroids. By mixing HA, Collagen I, and Matrigel, we optimized and obtained BSM matrix gel that resembled the brain extracellular components in terms of biochemical composition and elastic modulus. We applied the 3D culture and analysis system to assess the invasion capacity of GBM cells by embedding the GBM multicellular spheroids in the BSM matrix gels followed by high-content imaging. We compared the invasion capacity of DBTRG and U251 GBM cell lines through multiple quantitative parameters acquired from the time-lapse imaging data and revealed that the invasion capacity of DBTRG was higher than that of U251. Our mechanistic study disclosed several potential pathways and genes that might play an important role in regulating GBM invasion. Our system provided a useful platform for the analysis of GBM invasion in a 3D medium that mimics brain extracellular matrix, which can facilitate the mechanistic study and the development of novel therapeutic strategy of GBM.

## Data Availability

The datasets presented in this study can be found in online repositories. The names of the repository/repositories and accession number(s) can be found at: https://www.ncbi.nlm.nih.gov/geo/, GSE200574.
