# Supplementary figures and images for: Novel Brain-Stiffness-Mimicking Matrix Gel Enables Comprehensive Invasion Analysis of 3D Cultured GBM Cells
Source: Front Mol Biosci. 2022 Jun 9;9:885806. doi: 10.3389/fmolb.2022.885806 (PMC9218788; doi:10.3389/fmolb.2022.885806)

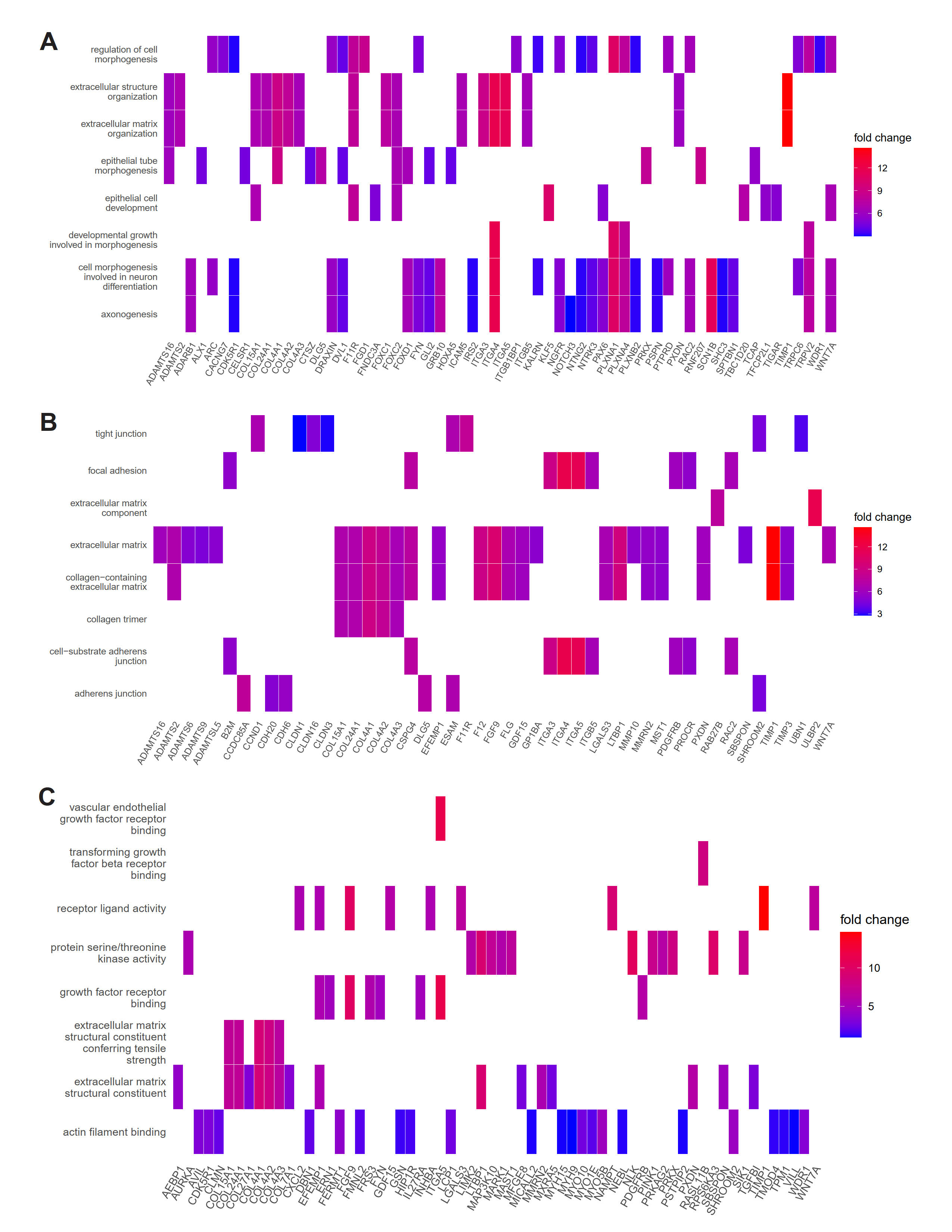

Supplement: Supplementary file 1 [file Image3.jpg]

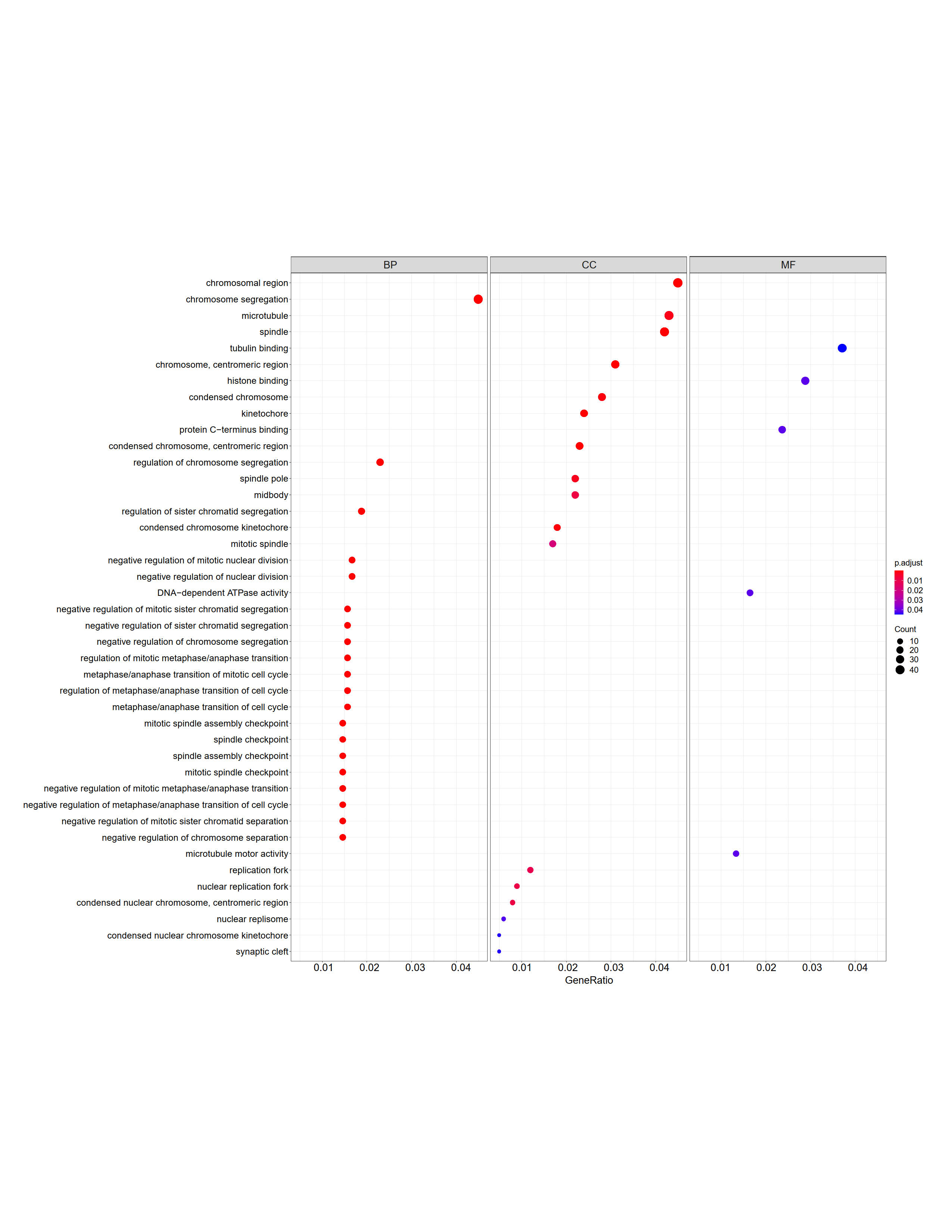

Supplement: Supplementary file 2 [file Image2.jpg]

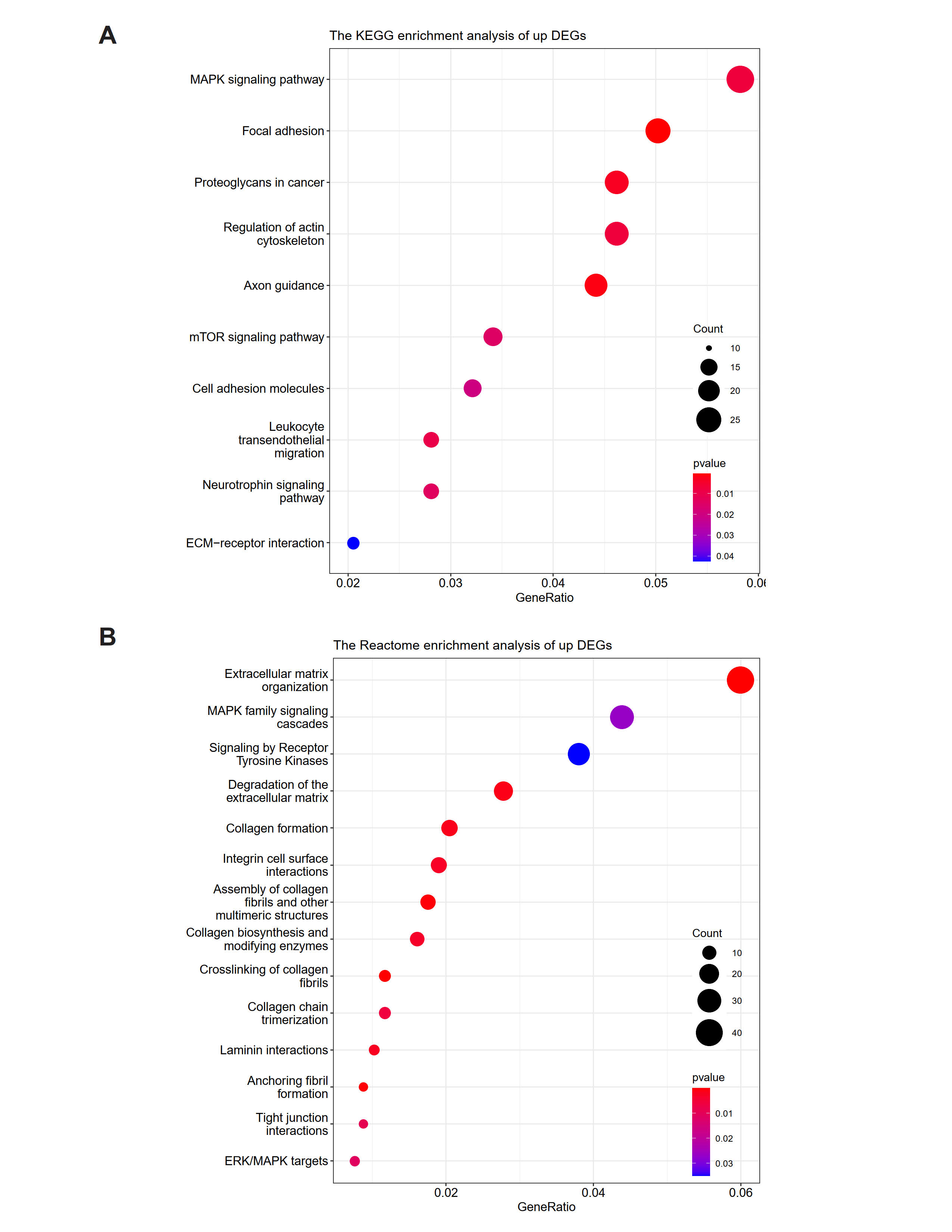

Supplement: Supplementary file 4 [file Image4.jpg]

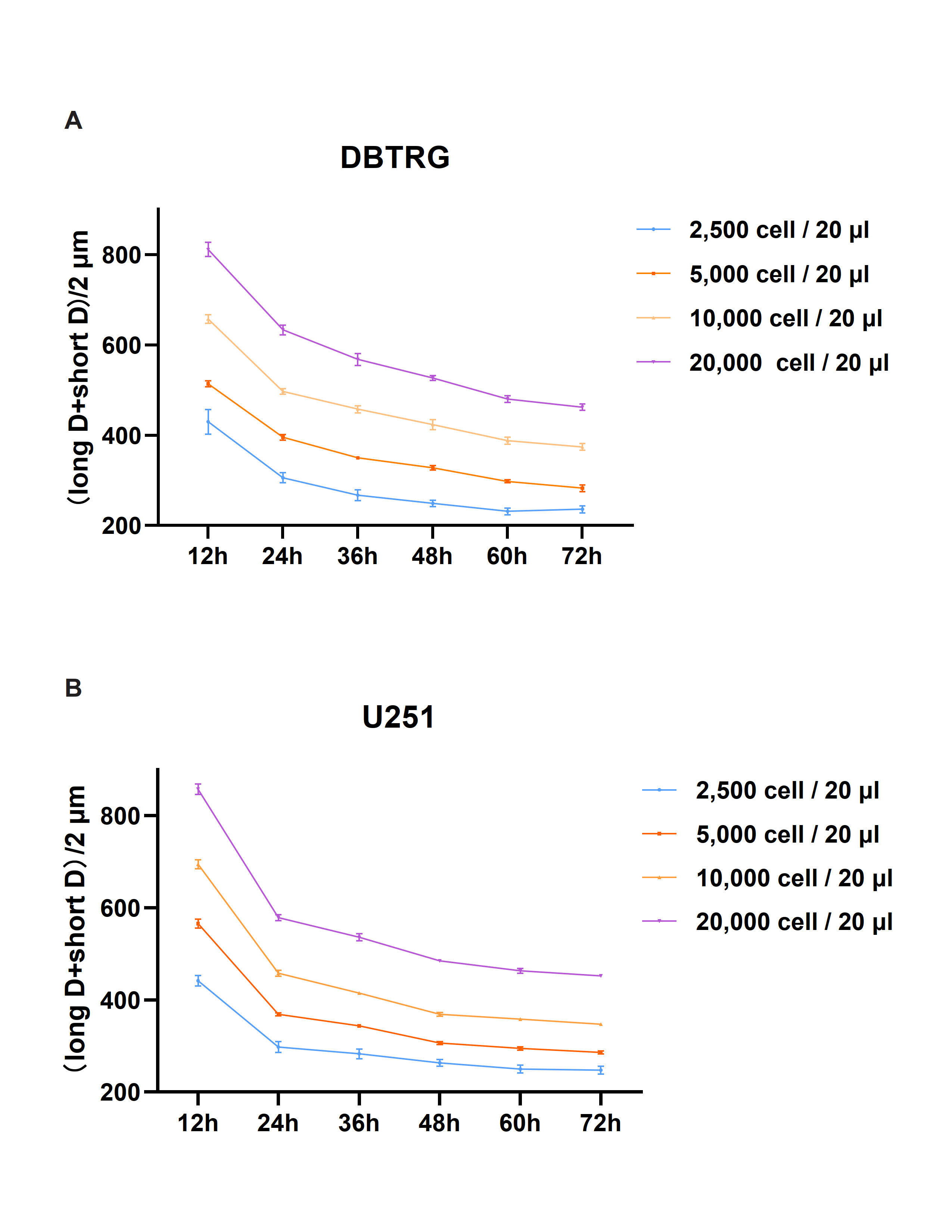

Supplement: Supplementary file 5 [file Image1.jpg]
